# Supplementary material for: Molecular mechanism establishing the OFF pathway in vision
Source: Nat Commun. 2025 Apr 18;16:3708. doi: 10.1038/s41467-025-59046-0 (PMC12008213; doi:10.1038/s41467-025-59046-0)
Supplement: Supplementary file 1 — Supplementary Information [file 41467_2025_59046_MOESM1_ESM.pdf]

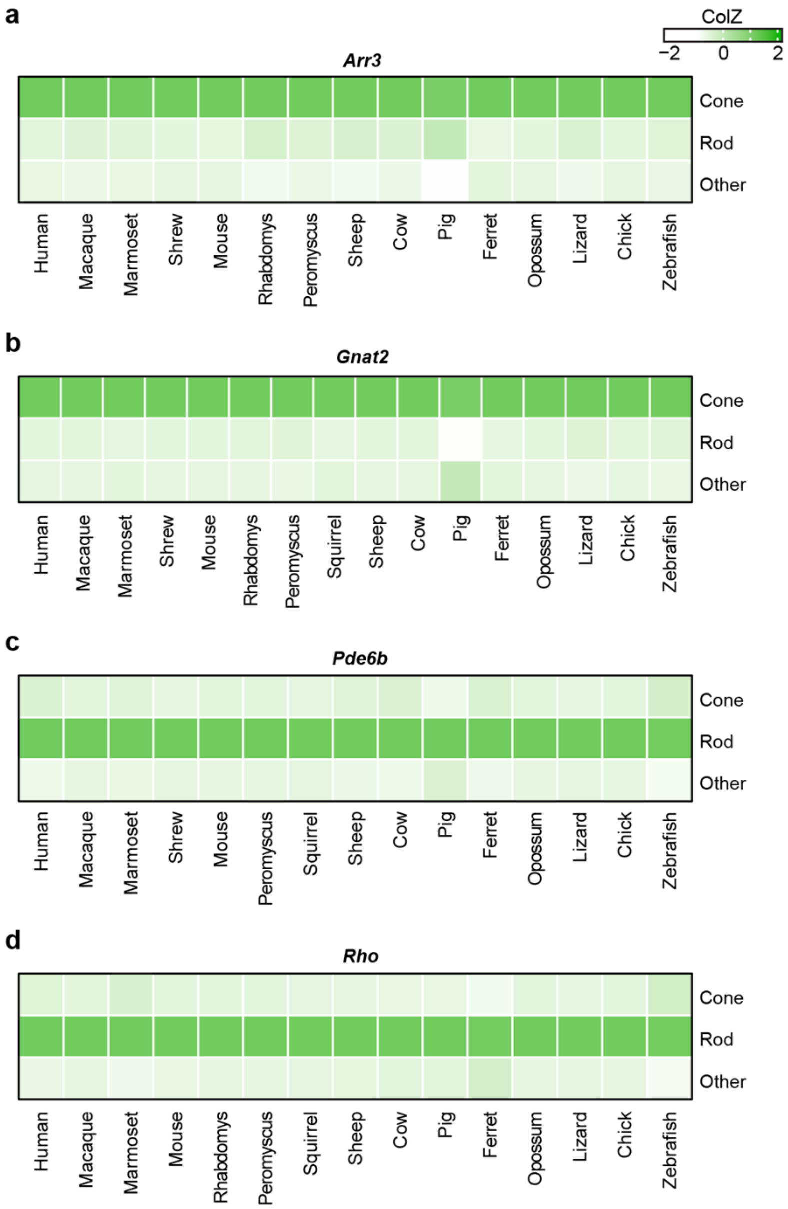

Supplementary Fig. 1 | Cone and rod marker gene expression across species. a Heatmap of z-scores (by column) of scRNA-seq data [1] comparing expression of *Arr3*, a cone marker gene across species. **b-d** Analogous to a for additional cone (b, *Gnat*) and rod (c and d, *Pde6b* and *Rh*) marker genes.

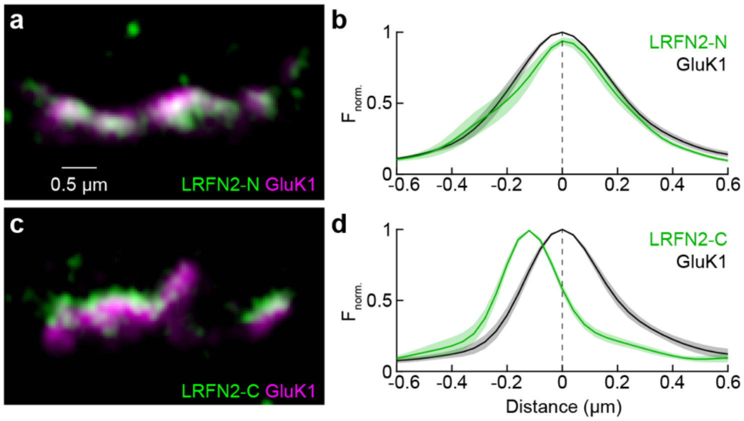

Supplementary Fig. 2 | Stratification of LRFN2 N- vs. C-terminal staining indicates expression in cones. a Representative super-resolution images of the OPL in wild-type retinas stained with antibodies against the N-terminus of LRFN2 (*green*) and GluK1 (*magenta*). **b** Normalized fluorescence intensity profiles of this LRFN2 staining (*green*) perpendicular through the OPL centered on the peak of GluK1 (*black*, n = 10 cones from 3 retinas). **c, d** Analogous to c, d for staining with an antibody against the C-terminus of LRFN2 (c, *green*; d, *green*) and the same antibody as in a, b against GluK1 (c, *magenta*, d, *black*, n = 7 cones from 2 retinas). The upward shift of the labeling for the intracellular C-terminus vs. the extracellular N-terminus is consistent with a localization of LRFN2 in cones.

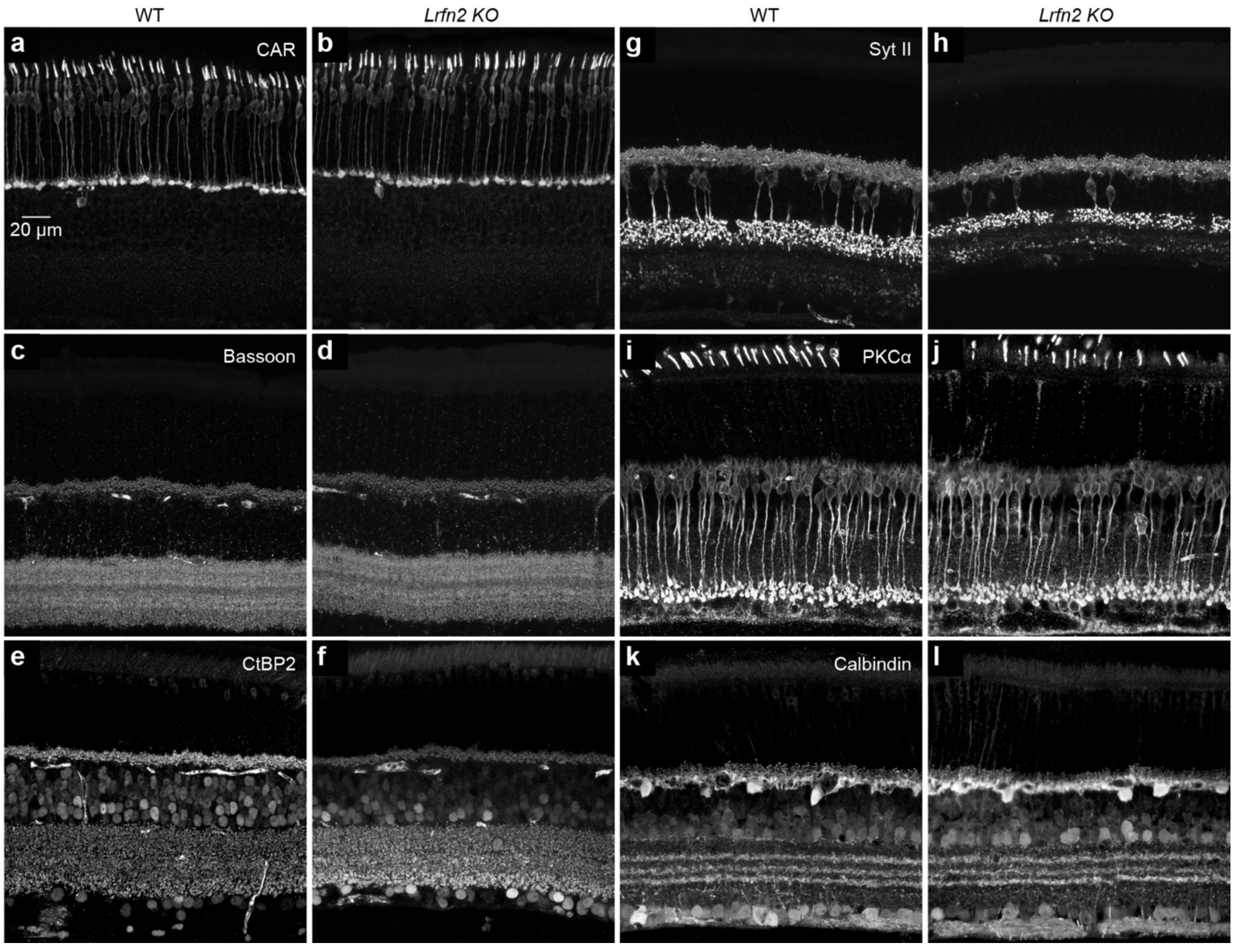

Supplementary Fig. 3 | Normal soma distributions and neurite stratification patterns in *Lrfn2 KO* mice. a, b Representative images of retinal vibratome sections from wild-type (A) and *Lrfn2 KO* (B) mice stained for CAR. **c-l** Analogous to a, b for staining for Bassoon (c, d), CtBP2 (e, f), Syt II (g, h), PKCα (i, j), and Calbindin (k, l).

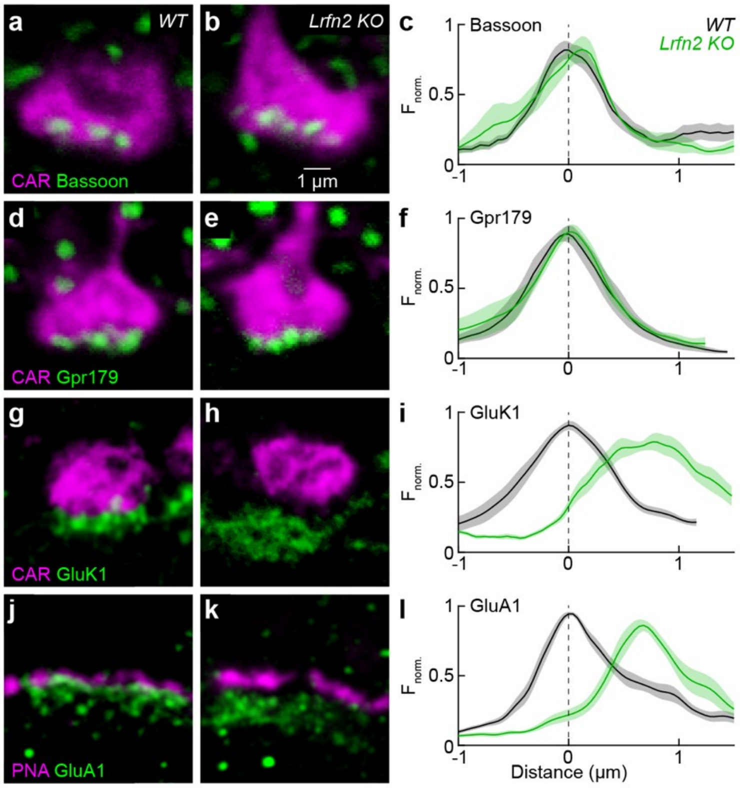

Supplementary Fig. 4 | LRFN2 organizes glutamate receptors in the OFF bipolar cell postsynapse. **a, b** Representative super-resolution images of cone pedicles stained for CAR (*magenta*) and Bassoon (*green*) in wild-type (a) and *Lrfn2 KO* (b) retinas. **c** Normalized fluorescence intensity profiles of the Bassoon staining in wild-type (*black*, n = 8 cones from 3 retinas) and *Lrfn2 KO* (*green*, n = 6 cones from 2 retinas) mice centered on the lower position of 25% of the maximal CAR amplitude. **d-f** Analogous to a-c for staining for Gpr179 (*green*) in wild-type (n = 8 cones from 2 retinas) and *Lrfn2 KO* (n = 8 cones from 3 retinas) mice. **g-i** Analogous to a-c for staining for GluK1 (*green*) in wild-type (n = 15 cones from 4 retinas) and *Lrfn2 KO* (n = 10 cones from 3 retinas) mice. **j-l** Analogous to a-c for staining for GluA1 (*green*) in wild-type (n = 10 cones from 3 retinas) and *Lrfn2 KO* (n = 10 cones from 3 retinas) mice. Normalized fluorescence intensity profiles here are centered on the peak of the PNA (*magenta*) profiles.

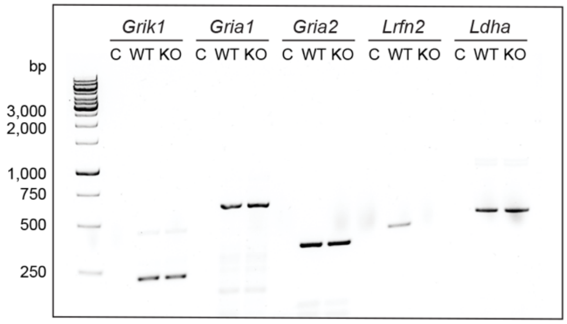

Supplementary Fig. 5 | RT-PCR analysis of glutamate receptor expression in wild-type and *Lrfn2 KO* retinas. Total RNA was extracted from retinas (wild-type [WT] or *Lrfn2 KO* [KO]) using the RNeasy kit (Qiagen). For each sample, 300 µg of total RNA was reverse-transcribed in separate reactions primed with oligo (dT) 12–18 or random hexamers (SuperScript™ IV First-Strand Synthesis System, Invitrogen), and the resulting cDNAs were combined. Two microliters of each cDNA pool were used for PCR amplification (KlenTaq polymerase). Five microliters of each PCR reaction were resolved by agarose gel electrophoresis. Gene targets are indicated at the top: *Grik1* encodes GluK1, *Gria1* encodes GluA1, and *Gria2* encodes GluA2. 'C' denotes the control (no template). The molecular weight marker is the 1 kb GeneRuler™ (Thermo Fisher Scientific), and band sizes (in base pairs) are shown on the left.

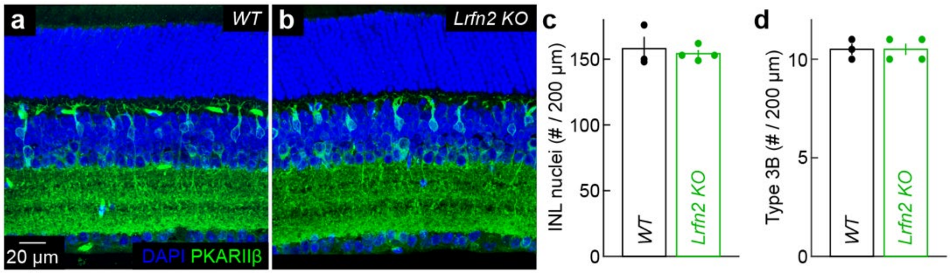

Supplementary Fig. 6 | Bipolar cell density is unchanged in *Lrfn2 KO* mice. a, b Representative images of vibratome sections through wild-type (a) and *Lrfn2 KO* (b) retinas, in which nuclei are labeled by DAPI (*blue*) and type 3B OFF bipolar cells, in addition to a subset of amacrine cells, are stained for PKARIIβ (*green*). c, d Summary data of the density of DAPI-stained nuclei in the INL (c, wild-type, *black*: n = 3 mice, *Lrfn2 KO*, *green*: n = 4 mice, p = 0.8 by two-sided Mann-Whitney *U* test) and PKARIIβ-positive bipolar cells (d, wild-type, *black*: n = 3 mice, *Lrfn2 KO*, *green*: n = 4 mice, p = 1 by two-sided Mann-Whitney *U* test).

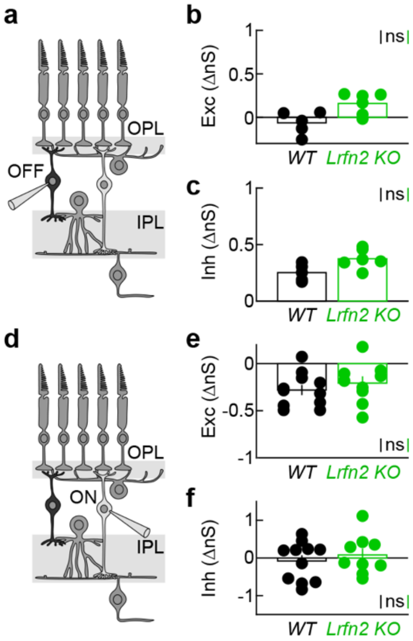

Supplementary Fig. 7 | Bipolar cell synaptic inputs in *Lrfn2 KO* mice. a Schematic illustrating the retinal circuitry and OFF bipolar patch-clamp recordings. The schematic shows a cross-sectional retina view; OFF bipolar cells were targeted in retinal flat mounts. **b, c** Summary data of the peak excitatory (b, wild-type, *black*: n = 5 cells from 5 retinas, *Lrfn2 KO*, *green*: n = 6 cells from 6 retinas, p = 0.052 by two-sided Mann-Whitney *U* test) and inhibitory synaptic conductances (c, wild-type, *black*: n = 5 cells from 5 retinas, *Lrfn2 KO*, *green*: n = 6 cells from 6 retinas, p = 0.052 by two-sided Mann-Whitney *U* test) during the stimulus ON and OFF phase, respectively. **d** Schematic illustrating the retinal circuitry and ON bipolar patch-clamp recordings. **e, f** Summary data of the peak excitatory (e, wild-type, *black*: n = 11 cells from 11 retinas, *Lrfn2 KO*, *green*: n = 9 cells from 9 retinas, p = 0.36 by two-sided Mann-Whitney *U* test) and inhibitory synaptic conductances (f, wild-type, *black*: n = 11 cells from 11 retinas, *Lrfn2 KO*, *green*: n = 9 cells from 9 retinas, p = 0.54 by two-sided Mann-Whitney *U* test) during the stimulus OFF and ON phase, respectively.

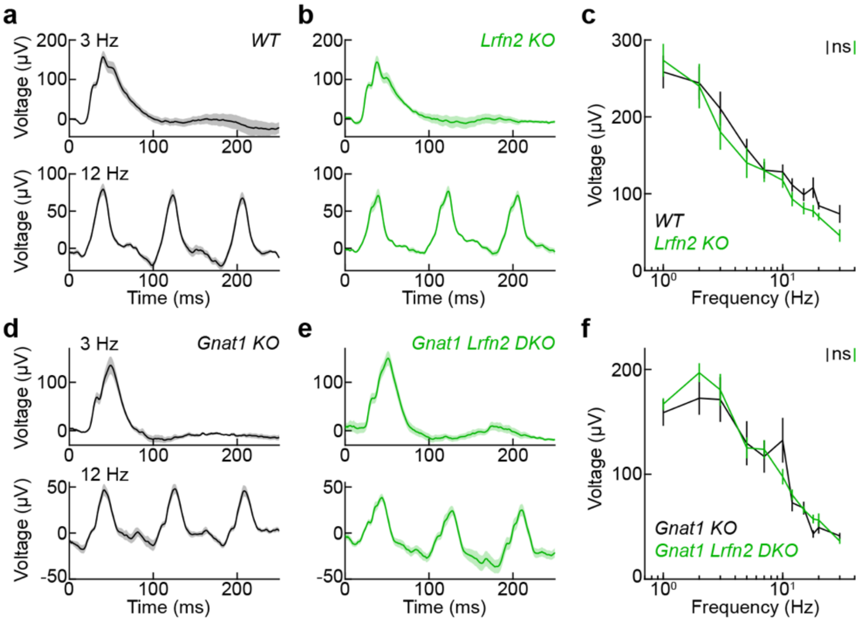

Supplementary Fig. 8 | *Lrfn2 KO* does not affect flicker ERG responses. a, b Traces (shaded areas) indicate mean (± SEM) flicker ERG responses of wild-type (a, *black*) and *Lrfn2 KO* (b, *green*) mice to 3 Hz (*top panels*) and 12 **c** Summary data of flicker ERG responses from wild-type (n = 10 mice) and *Lrfn2 KO* (n = 8 mice, p = 0.58 by two-sided bootstrap test) animals. **d-f** Analogous to a-c for recordings on a *Gnat1 KO* background eliminating rod-driven light responses (*Gnat1 KO*, *black*: n = 10, *Gnat1 Lrfn2 DKO*, *green*: n = 8 mice, p = 0.43 by two-sided bootstrap test).

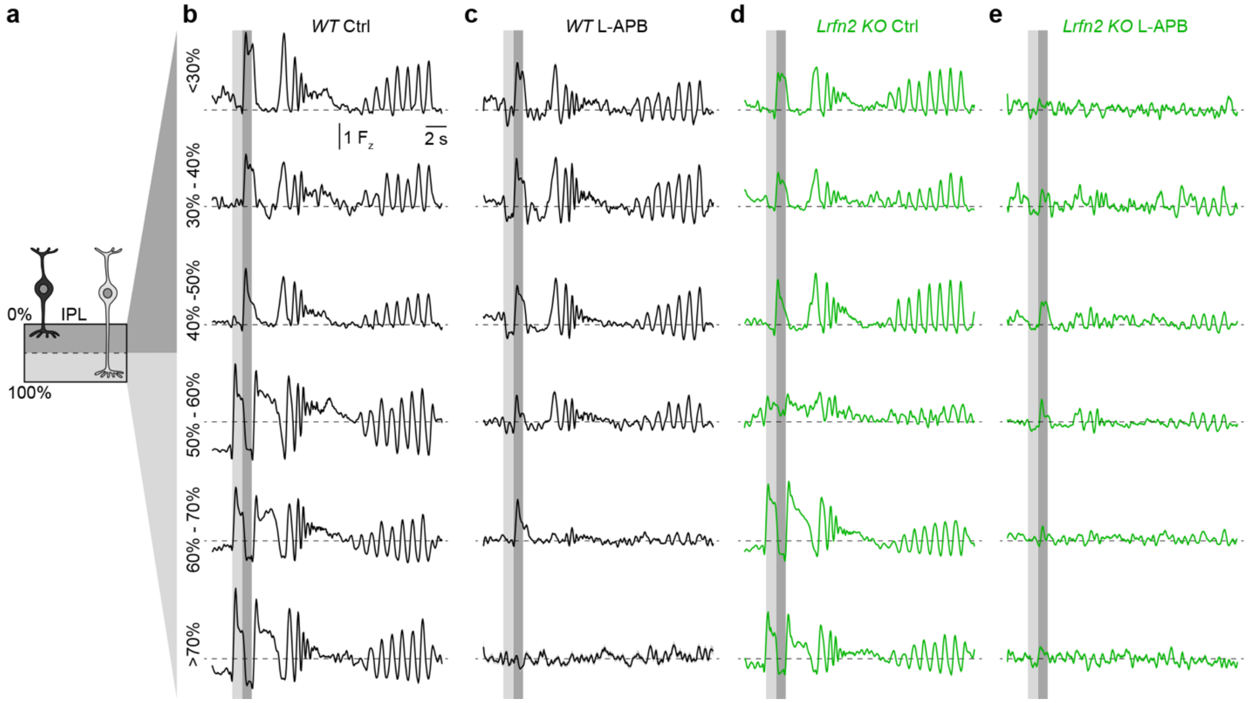

Supplementary Fig. 9 | Glutamate release across the IPL. a Schematic illustrating the axonal stratification of ON and OFF bipolar cells in the IPL. **b-e** Mean (± SEM) traces of ROI responses to the chirp stimulus for the top 50% (in terms of repeat reliability) at each depth in wild-type mice (*black*) in control conditions (b, <30%: n = 153, 30%-40%: n = 63, 40%-50%: n = 247, 50%-60%: n = 97, 60%-70%: n = 185, >70%: n = 90) and the presence of L-APB (c, <30%: n = 92, 30%-40%: n = 95, 40%-50%: n = 310, 50%-60%: n = 155, 60%-70%: n = 158, >70%: n = 30) and *Lrfn2 KO* mice (*green*) in control conditions (d, <30%: n = 212, 30%-40%: n = 362, 40%-50%: n = 427, 50%-60%: n = 219, 60%-70%: n = 462, >70%: n = 215) and the presence of L-APB (e, <30%: n = 90, 30%-40%: n = 124, 40%-50%: n = 273, 50%-60%: n = 444, 60%-70%: n = 245, >70%: n = 89). ON and OFF steps at the beginning of the stimulus are shaded in dark and light gray, respectively. Rows summarize responses of ROIs at different depths as indicated on the left.

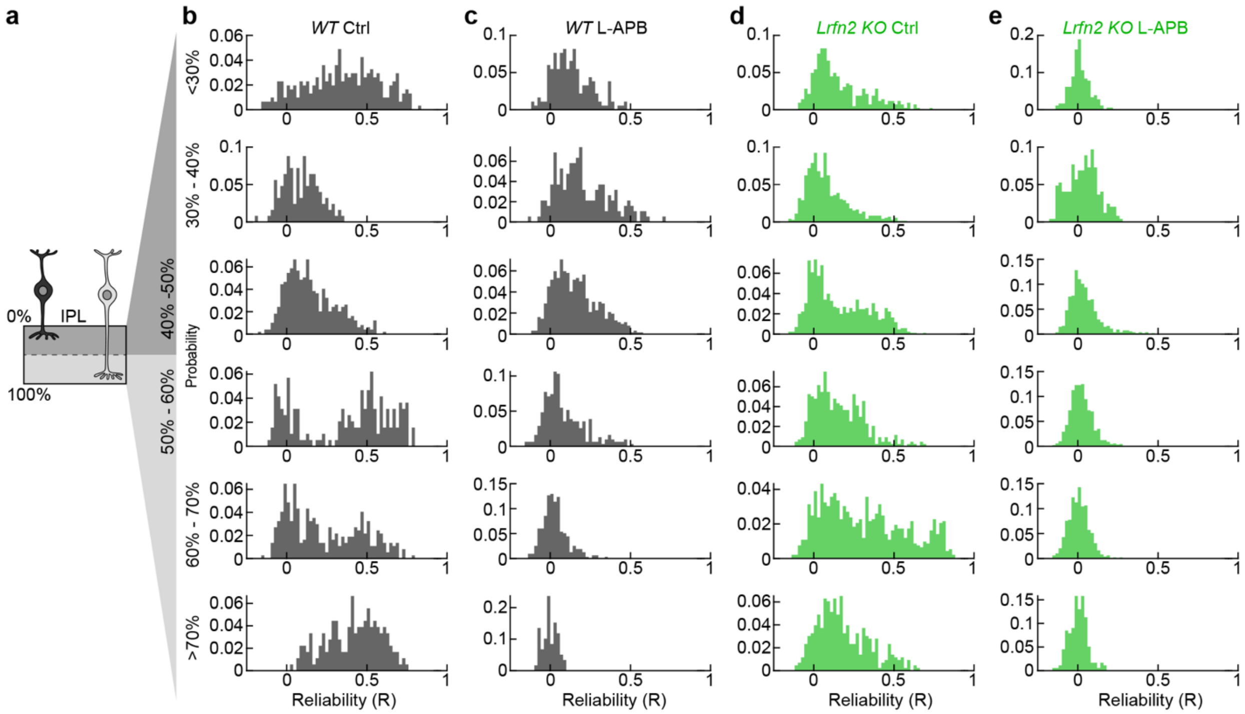

Supplementary Fig. 10 | Reliability of light-evoked glutamate release across the IPL. a Schematic illustrating the axonal stratification of ON and OFF bipolar cells in the IPL. **b-e** Distributions of the repeat reliability of ROI responses to chirp stimuli for the top 50% of ROIs (in terms of repeat reliability) at each depth in wild-type mice (*black*) in control conditions (b) and the presence of L-APB (c) and *Lrfn2 KO* mice (*green*) in control conditions (d) and the presence of L-APB. Throughout this figure, n's are identical to Supplementary Fig. 9.

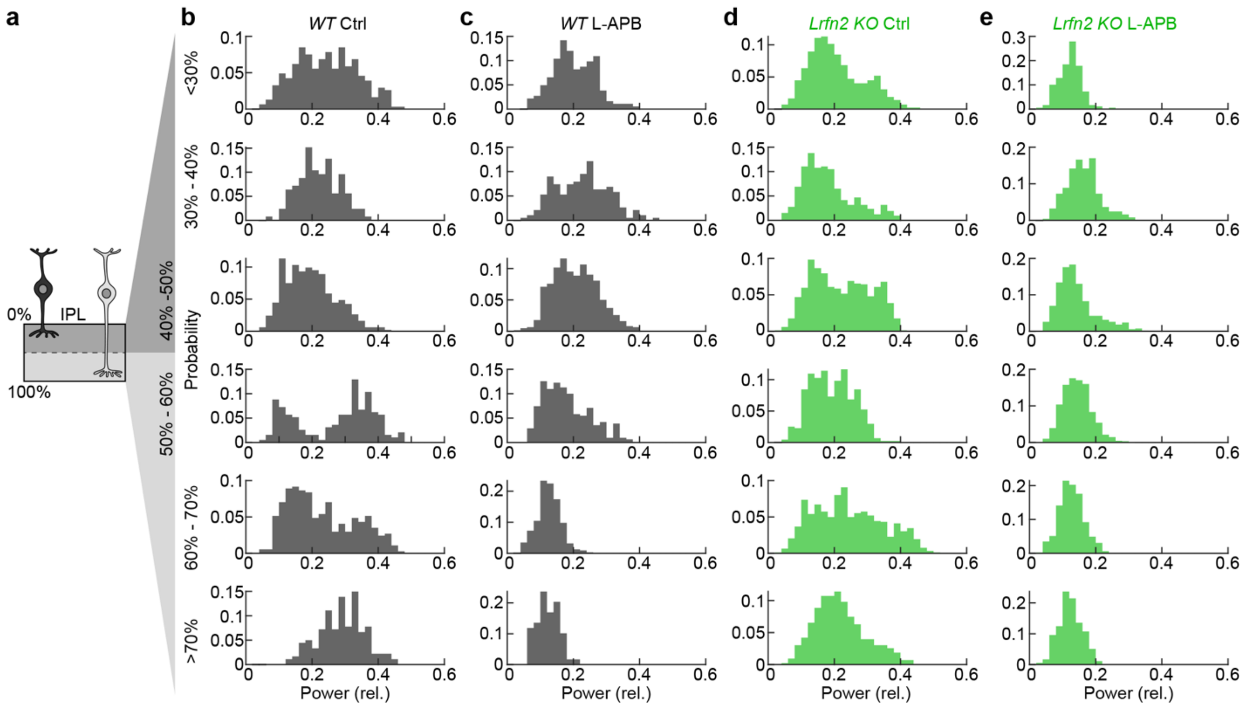

Supplementary Fig. 11 | Frequency-entrained response power of glutamate release across the IPL. a Schematic illustrating the axonal stratification of ON and OFF bipolar cells in the IPL. **b-e** Distributions of the relative power of ROI responses (top 50% of ROIs in terms of repeat reliability at each depth) at the stimulus frequency during the final chirp stimulus segment in which contrast gradually increases at a fixed temporal frequency (1 Hz) in wild-type mice (*black*) in control conditions (b) and the presence of L-APB (c) and *Lrfn2 KO* mice (*green*) in control conditions (d) and the presence of L-APB. Throughout this figure, n's are identical to Supplementary Fig. 9.

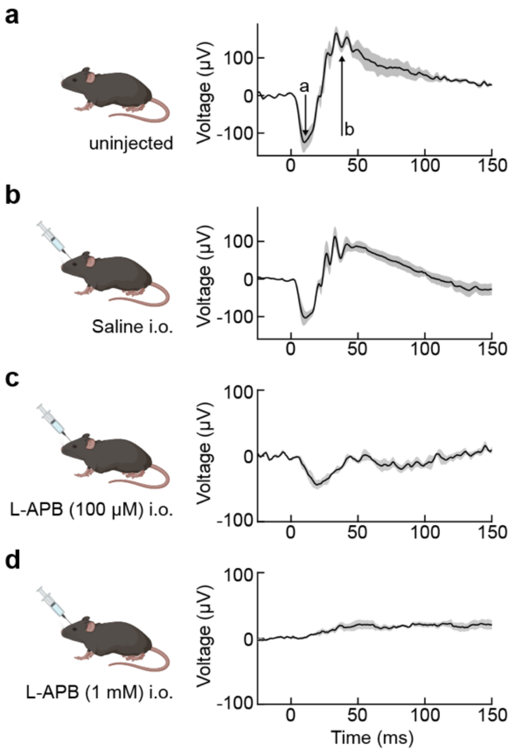

Supplementary Fig. 12 | Intraocular L-APB injections and ERG responses. a Traces (shaded areas) indicate mean (± SEM) ERG responses to a photopic flash (time = 0) recorded from uninjected wild-type mice (n = 3 mice). **b-d** Analogous to a for recordings from wild-type mice intraocularly injected with saline (b, n = 4 mice), 100 µM L-APB (c, n = 3 mice), and 1 mM L-APB (d, n = 6 mice). Schematics on the left created in BioRender. Kerschensteiner, D. (2025) https://BioRender.com/y70x093

References cited in the Supplementary information

1. Hahn J, *et al.* Evolution of neuronal cell classes and types in the vertebrate retina. *Nature* **624**, 415-424 (2023).
