# Supplementary material for: Molecular mechanism establishing the OFF pathway in vision
Source: Nat Commun. 2025 Apr 18;16:3708. doi: 10.1038/s41467-025-59046-0 (PMC12008213; doi:10.1038/s41467-025-59046-0)
Supplement: Supplementary file 2 — Reporting Summary [file 41467_2025_59046_MOESM2_ESM.pdf]

nature portfolio

Corresponding author(s): Daniel Kerschensteiner

Last updated by author(s): Jan 28, 2025

Reporting Summary

Nature Portfolio wishes to improve the reproducibility of the work that we publish. This form provides structure for consistency and transparency in reporting. For further information on Nature Portfolio policies, see our Editorial Policies and the Editorial Policy Checklist.

Statistics

For all statistical analyses, confirm that the following items are present in the figure legend, table legend, main text, or Methods section.

n/a	Confirmed	
☐	☒	The exact sample size (*n*) for each experimental group/condition, given as a discrete number and unit of measurement
☐	☒	A statement on whether measurements were taken from distinct samples or whether the same sample was measured repeatedly
☐	☒	The statistical test(s) used AND whether they are one- or two-sided<br>*Only common tests should be described solely by name; describe more complex techniques in the Methods section.*
☒	☐	A description of all covariates tested
☐	☒	A description of any assumptions or corrections, such as tests of normality and adjustment for multiple comparisons
☐	☒	A full description of the statistical parameters including central tendency (e.g. means) or other basic estimates (e.g. regression coefficient) AND variation (e.g. standard deviation) or associated estimates of uncertainty (e.g. confidence intervals)
☐	☒	For null hypothesis testing, the test statistic (e.g. *F*, *t*, *r*) with confidence intervals, effect sizes, degrees of freedom and *P* value noted<br>*Give P values as exact values whenever suitable.*
☒	☐	For Bayesian analysis, information on the choice of priors and Markov chain Monte Carlo settings
☒	☐	For hierarchical and complex designs, identification of the appropriate level for tests and full reporting of outcomes
☒	☐	Estimates of effect sizes (e.g. Cohen's *d*, Pearson's *r*), indicating how they were calculated

Our web collection on statistics for biologists contains articles on many of the points above.

Software and code

Policy information about availability of computer code

Data collection	Super-resolution images were acquired using ZEN Microscopy Software (Zeiss). Patch-clamp electrophysiology data were acquired using Axon pClamp 10 (Molecular Devices. Two-photon imaging series (z-stacks and time series) were acquired using the ScanImage r3.8 toolbox in MATLAB. Electroretinograms were recorded on a TAS Visual Electrodiagnostic Testing System (LKC Technologies). The behavioral responses of mice to looming stimuli were recorded with a USB camera (Logitech) and Windows Movie Maker (Microsoft).
Data analysis	Anatomical imaging data were analyzed in Amira (Visage Imaging), ImageJ/Fiji (NIH), and custom scripts written in MATLAB (The Mathworks). Behavioral data were tracked with ANY-maze (Stoelting). Functional imaging data, patch-clamp recordings, and electroretinograms were analyzed using custom scripts written in MATLAB. Custom scripts used in this study are available at https://github.com/kerschensteinerd/soto_natcommun_2025. Adobe Illustrator 2025 (Version 29.2.1) was used to assmble the final figures.

For manuscripts utilizing custom algorithms or software that are central to the research but not yet described in published literature, software must be made available to editors and reviewers. We strongly encourage code deposition in a community repository (e.g. GitHub). See the Nature Portfolio guidelines for submitting code & software for further information.

Data

Policy information about availability of data

All manuscripts must include a data availability statement. This statement should provide the following information, where applicable:

- Accession codes, unique identifiers, or web links for publicly available datasets
- A description of any restrictions on data availability
- For clinical datasets or third party data, please ensure that the statement adheres to our policy

Source data, including all preprocessed information for analyses, traces, and graphs in figures is provided in a spreadsheet. Functional two-photon imaging data are additionally included as a MATLAB file in the GitHub repository for this paper (https://github.com/kerschensteinerd/soto_natcommun_2025).

Research involving human participants, their data, or biological material

Policy information about studies with human participants or human data. See also policy information about sex, gender (identity/presentation), and sexual orientation and race, ethnicity and racism.

Reporting on sex and gender	*Use the terms sex (biological attribute) and gender (shaped by social and cultural circumstances) carefully in order to avoid confusing both terms. Indicate if findings apply to only one sex or gender; describe whether sex and gender were considered in study design; whether sex and/or gender was determined based on self-reporting or assigned and methods used.* *Provide in the source data disaggregated sex and gender data, where this information has been collected, and if consent has been obtained for sharing of individual-level data; provide overall numbers in this Reporting Summary. Please state if this information has not been collected.* *Report sex- and gender-based analyses where performed, justify reasons for lack of sex- and gender-based analysis.*
Reporting on race, ethnicity, or other socially relevant groupings	*Please specify the socially constructed or socially relevant categorization variable(s) used in your manuscript and explain why they were used. Please note that such variables should not be used as proxies for other socially constructed/relevant variables (for example, race or ethnicity should not be used as a proxy for socioeconomic status).* *Provide clear definitions of the relevant terms used, how they were provided (by the participants/respondents, the researchers, or third parties), and the method(s) used to classify people into the different categories (e.g. self-report, census or administrative data, social media data, etc.)* *Please provide details about how you controlled for confounding variables in your analyses.*
Population characteristics	*Describe the covariate-relevant population characteristics of the human research participants (e.g. age, genotypic information, past and current diagnosis and treatment categories). If you filled out the behavioural & social sciences study design questions and have nothing to add here, write "See above."*
Recruitment	*Describe how participants were recruited. Outline any potential self-selection bias or other biases that may be present and how these are likely to impact results.*
Ethics oversight	*Identify the organization(s) that approved the study protocol.*

Note that full information on the approval of the study protocol must also be provided in the manuscript.

Field-specific reporting

Please select the one below that is the best fit for your research. If you are not sure, read the appropriate sections before making your selection.

☒ Life sciences ☐ Behavioural & social sciences ☐ Ecological, evolutionary & environmental sciences

For a reference copy of the document with all sections, see nature.com/documents/nr-reporting-summary-flat.pdf

Life sciences study design

All studies must disclose on these points even when the disclosure is negative.

Sample size	We did not perform power analyses to determine the sample size for each experiment. Instead sample sizes were selected to reflect the standard in the field, including our previous studies (e.g., Soto et al., 2022 and Soto et al., 2018).
Data exclusions	Mice were only excluded from analyses if their eyes had been damaged by intraocular injections. In these cases, no experiments were performed and therefore no data were excluded.
Replication	Our findings were replicated across mice from different litters. Different methods (patch-clamp recordings, two-photon glutamate imaging, super-resolution anatomy) support our conclusions about the role of LRFN2 in the formation of the retinal OFF pathway. Data collection spanned approximately two years with no noticeable differences in the results.
Randomization	In functional experiments, visual stimuli were shown in pseudorandom orders.
Blinding	For patch-clamp recordings, two-photon glutamate imaging, electroretinography, and behavioral experiments, the experimenter was blinded

Blinding	to the genotype of the animal.

Reporting for specific materials, systems and methods

We require information from authors about some types of materials, experimental systems and methods used in many studies. Here, indicate whether each material, system or method listed is relevant to your study. If you are not sure if a list item applies to your research, read the appropriate section before selecting a response.

Materials & experimental systems

n/a	Involved in the study
☐	☒ Antibodies
☒	☐ Eukaryotic cell lines
☒	☐ Palaeontology and archaeology
☐	☒ Animals and other organisms
☒	☐ Clinical data
☒	☐ Dual use research of concern
☒	☐ Plants

Methods

n/a	Involved in the study
☒	☐ ChIP-seq
☒	☐ Flow cytometry
☒	☐ MRI-based neuroimaging

Antibodies

Antibodies used	mouse anti-PKC (Sigma, 1:500, RRID: AB_477375), mouse anti-CtBP2 (BD Biosciences, 1:500, RRID:AB_399431), chicken anti-GFP (Invitrogen, 1:1000, RRID:AB_770014), rabbit anti-GluA1 (Millipore, 1:500, RRID:AB_2721164), rabbit anti-LRFN2 (against N-terminus, 1:1000), rabbit anti-LRFN2 (against C-terminus, Sigma, 1:1000, RRID:AB_2138711), mouse anti-Bassoon (Abcam, 1:200, RRID: AB_1860018), mouse anti-Dystrophin (Santa Cruz, 1:250, RRID: AB_1122390), mouse anti-GluA2 (Invitrogen, 1:200, RRID: AB_2533058), mouse anti-GluK1 (Santa Cruz, 1:100, RRID: AB-2716684), mouse anti-Synaptotagmin 2 (Developmental Studies Hybridoma Bank, 1:200, RRID: AB_2315626), sheep anti-mGluR6 (1:500, kind gift of Dr. Martemyanov), Alexa 488-conjugated anti-chicken IgY anti-rabbit IgG, and Alexa 568-conjugated anti-mouse IgG, anti-goat IgG, and anti-sheep IgG (Invitrogen).
Validation	Commercial antibodies were verified by the manufacturers as described on their information. In addition all primary antibodies used in this study have been used frequently on the retina, including by us (e.g., Soto et al., 2020, Soto et al. 2018, Soto et al. 2013). The specificity of both LRFN2 antibodies was also confirmed in this study using Lrfn2 KO mice.

Animals and other research organisms

Policy information about studies involving animals; ARRIVE guidelines recommended for reporting animal research, and Sex and Gender in Research

Laboratory animals	All laboratory animals used in this study, mice, were maintained in a colony within the Division of Comparative Medicine at Washington University School of Medicine. All mice were crossed onto the C57BL/6J background for at least five generations. Animals were housed on a 12h/12h light/dark cycle with overnight dark adaptation before all experiments. Mice had ad libidum access to food and water. Experiments were conducted at 20-21 °C with humidity levels kept between 30% and 50%. Intravitreal injections were performed at postnatal day 1 to 6.
Wild animals	This study did not involve wild animals.
Reporting on sex	Experiments utilized adult mice of both sexes, with an almost equal distribution. No significant differences were noted in the results related to the sex of the animals.
Field-collected samples	This study did not involve samples collected from the field.
Ethics oversight	All procedures were approved by the Institutional Animal Care and Use Committee of Washington University School of Medicine and complied with the National Institutes of Health Guide for the Care and Use of Laboratory Animals.

Note that full information on the approval of the study protocol must also be provided in the manuscript.

Plants

Seed stocks

Report on the source of all seed stocks or other plant material used. If applicable, state the seed stock centre and catalogue number. If plant specimens were collected from the field, describe the collection location, date and sampling procedures.

Novel plant genotypes

Describe the methods by which all novel plant genotypes were produced. This includes those generated by transgenic approaches, gene editing, chemical/radiation-based mutagenesis and hybridization. For transgenic lines, describe the transformation method, the number of independent lines analyzed and the generation upon which experiments were performed. For gene-edited lines, describe the editor used, the endogenous sequence targeted for editing, the targeting guide RNA sequence (if applicable) and how the editor was applied.

Authentication

Describe any authentication procedures for each seed stock used or novel genotype generated. Describe any experiments used to assess the effect of a mutation and, where applicable, how potential secondary effects (e.g. second site T-DNA insertions, mosiacism, off-target gene editing) were examined.
